# Supplementary material for: The self-management support needs of people diagnosed with psoriatic arthritis: a realist review protocol
Source: BMJ Open. 2026 Feb 2;16(2):e110531. doi: 10.1136/bmjopen-2025-110531 (PMC12878256; doi:10.1136/bmjopen-2025-110531)
Supplement: online supplemental file 2 [file bmjopen-16-2-s002.docx]

**Summary of papers found by initial search – October 2024**

Nine papers reviewed following an initial search of CINAHL, Medline and APA PsychInfo (EBSCO host)

| **FIRST AUTHOR** | **YEAR** | **TITLE** | **KEY FINDINGS** |
| --- | --- | --- | --- |
| Lebwohl, M.(1) | 2022 | Evolution of Patient Perceptions of Psoriatic Disease: Results from the Understanding Psoriatic Disease Leveraging Insights for Treatment (UPLIFT) Survey | - Patients with at least one affected special area (scalp, face, genitalia, palms/nails, soles) reported lower QoL - Factors that define disease severity for patients are type of symptoms and disease duration - Factors that define disease severity for clinicians are quality of life and amount of skin involvement - Patients find most treatment options (for psoriasis) burdensome |
| Merola, J.F.(2) | 2023 | Patient and physician perceptions of psoriatic disease in the United States: Results from the UPLIFT study | - Most patients with PsA felt they were not too closely (32.1%) or not at all (59.3%) aligned with their rheumatologist regarding treatment goals. - Most patients reported a moderate (31.8%) or strong (53.9%) need for better treatments |
| Eder, L.(3) | 2024 | Gender Differences in Perceptions of Psoriatic Arthritis Disease Impact, Management, and Physician Interactions: Results from a Global Patient Survey | - More women than men reported joint tenderness, skin plaques and enthesitis - More women than men reported major or moderate impact of PsA on their physical activity and emotional/mental wellbeing - More women were satisfied with their communication with HCPs and were more likely to discuss the impact of PsA on daily life, treatment satisfaction and treatment goals - The effect of PsA is greater on females - Men and women have different experiences with PsA |
| Candelas, G.(4) | 2016 | Benefit of health education by a training nurse in patients with axial and/or peripheral psoriatic arthritis: A systematic literature review | - Eight studies were included (5 RCTs) - Support from a trained nurse can increase rate of adherence to treatment prescribed by a rheumatologist, promotes self-management and increases patient satisfaction - Education programmes should include information on the diagnostic process, condition specific information, treatments, exercises, pain management and joint protection - Patients given the tools to develop in-depth knowledge of their condition are better be to cope with the physical and psychological challenges of PsA. |
| Dures, E.(5) | 2019 | Diagnosis and initial management in psoriatic arthritis: a qualitative study with patients | - Patients were highly distressed and anxious at the time of receiving their PsA diagnosis and dealing with functional limitations. - There is a need for guidelines on education, self-management and psychological support provision soon after diagnosis - HCPs need to work with patients to understand their current priorities and resources, to support self-management while avoiding blame or criticism. |
| Hong, C.(6) | 2022 | Patient and learner experience in a new set up of a multidisciplinary dermatology-rheumatology clinic care model for psoriatic arthritis | - The median rating of patient satisfaction was 8 out of 10, 96% of patients would “probably” or “definitely” recommend the service to others - Positive experiences of multidisciplinary clinic included time efficiency, seeing both specialties in one visit and thorough assessment - Negative experiences of multidisciplinary clinic included long waiting time, too many people in the room and lack of privacy |
| Ogdie, A.(7) | 2020 | Patient perspectives on the pathway to psoriatic arthritis diagnosis: results from a web-based survey of patients in the United States | - Misdiagnosis prior to receiving PsA diagnosis was common - More noticeable symptoms such as dactylitis and typical joint symptoms were associated with shorter times to diagnosis - Delays in diagnosis can lead to a greater rate in clinical progression and worse physical function |
| Bundy, N.(8) | 2023 | Self- evidence- based digital care programme improves health-related quality of life in adults with a variety of autoimmune diseases and long COVID: a retrospective study | - An evidence-based digital care programme that uses patient data to help identify hidden symptom triggers and guide personalised dietary and other non-pharmacological interventions was associated with a high level of engagement and adherence and statistically significant, clinically meaningful improvements in HRQoL - Patients with PsA demonstrated statistically significant improvements in social roles, ability to manage symptoms, physical function, anxiety, fatigue and pain interference. |
| Schaffer Blum, H.(9) | 2024 | Patients’ experience of a novel interdisciplinary nurse-led self-management intervention (INSELMA) - a qualitative evaluation | Four overall themes;   - New opportunity to improve symptoms and reduce long-held challenges that they had previously faced alone - Meaningful work with individual goals encompassing physical, social and psychological factors. - Empathy, support and coaching made participants aware of their own resources. Support from physio and OT with rheumatology experience was important - Most had decreased symptom load, increased self-management ability and expressed hope for the future. |

1. Lebwohl M, Langley RG, Paul C, Puíg L, Reich K, van de Kerkhof P, et al. Evolution of Patient Perceptions of Psoriatic Disease: Results from the Understanding Psoriatic Disease Leveraging Insights for Treatment (UPLIFT) Survey. Dermatol Ther (Heidelb). 2022 Jan 1;12(1):61–78.

2. Merola JF, Ogdie A, Gottlieb AB, Stein Gold L, Flower A, Jardon S, et al. Patient and Physician Perceptions of Psoriatic Disease in the United States: Results from the UPLIFT Survey. Dermatol Ther (Heidelb). 2023 June 1;13(6):1329–46.

3. Eder L, Richette P, Coates LC, Azevedo VF, Cappelleri JC, Johnson EP, et al. Gender Differences in Perceptions of Psoriatic Arthritis Disease Impact, Management, and Physician Interactions: Results from a Global Patient Survey. Rheumatol Ther. 2024 Oct 1;11(5):1115–34.

4. Candelas G, Villaverde V, García S, Guerra M, León MJ, Cañete JD. Benefit of health education by a training nurse in patients with axial and/or peripheral psoriatic arthritis: A systematic literature review. Rheumatol Int. 2016 Nov 1;36(11):1493–506.

5. Dures E, Bowen C, Brooke M, Lord J, Tillett W, McHugh N, et al. Diagnosis and initial management in psoriatic arthritis: a qualitative study with patients. Rheumatol Adv Pract. 2019;3(2):rkz022.

6. Hong C, Fang S, Yeo YW, Koh HY, Lee HY, Low AHL, et al. Patient and learner experience in a new set up of a multidisciplinary dermatology-rheumatology clinic care model for psoriatic arthritis. International Journal of Rheumatic Diseases. 2022;25(8):861–8.

7. Ogdie A, Nowell WB, Applegate E, Gavigan K, Venkatachalam S, de la Cruz M, et al. Patient perspectives on the pathway to psoriatic arthritis diagnosis: results from a web-based survey of patients in the United States. BMC Rheumatol. 2020;4:2.

8. Bundy N, De Jesus M, Lytle M, Calabrese L, Gobin C, Dyhrberg M. Self-evidence-based digital care programme improves health-related quality of life in adults with a variety of autoimmune diseases and long COVID: a retrospective study. RMD Open. 2023 May;9(2):e003061.

9. Blum NS, Esbensen BA, Østergaard M, Bremander A, Hendricks O, Lindgren LH, et al. Patients’ experience of a novel interdisciplinary nurse-led self-management intervention (INSELMA)-a qualitative evaluation. BMC Rheumatol. 2024 Mar 1;8(1):10.
