# Supplementary material for: The self-management support needs of people diagnosed with psoriatic arthritis: a realist review protocol
Source: BMJ Open. 2026 Feb 2;16(2):e110531. doi: 10.1136/bmjopen-2025-110531 (PMC12878256; doi:10.1136/bmjopen-2025-110531)
Supplement: online supplemental file 4 [file bmjopen-16-2-s004.pdf]

Fri, February 28, 2025 08:44:53 am

#	Query	Limiters/Expanders	Last Run Via	Results
S15	S6 AND S14	Limiters - Publication Date: 20100101-20261231; English Language<br>Expanders - Apply equivalent subjects<br>Search modes - Proximity	Interface - EBSCOhost Research Databases<br>Search Screen - Advanced Search<br>Database - CINAHL	122
S14	S7 OR S8 OR S9 OR S10 OR S11 OR S12 OR S13	Expanders - Apply equivalent subjects<br>Search modes - Proximity	Interface - EBSCOhost Research Databases<br>Search Screen - Advanced Search<br>Database - CINAHL	148,195
S13	(MH "Empowerment")	Expanders - Apply equivalent subjects<br>Search modes - Proximity	Interface - EBSCOhost Research Databases<br>Search Screen - Advanced Search<br>Database - CINAHL	19,394
S12	TI empower* OR AB empower*	Expanders - Apply equivalent subjects<br>Search modes - Proximity	Interface - EBSCOhost Research Databases<br>Search Screen - Advanced Search<br>Database - CINAHL	31,418
S11	(MH "Patient Education+")	Expanders - Apply equivalent subjects<br>Search modes - Proximity	Interface - EBSCOhost Research Databases<br>Search Screen - Advanced Search<br>Database - CINAHL	88,755
S10	TI "patient education" OR AB "patient education"	Expanders - Apply equivalent subjects<br>Search modes - Proximity	Interface - EBSCOhost Research Databases<br>Search Screen - Advanced Search<br>Database - CINAHL	13,762

S9	TI "self-management" OR AB "self-management"	Expanders - Apply equivalent subjects<br>Search modes - Proximity	Interface - EBSCOhost Research Databases<br>Search Screen - Advanced Search<br>Database - CINAHL	19,068
S8	(MH "Self-Management")	Expanders - Apply equivalent subjects<br>Search modes - Proximity	Interface - EBSCOhost Research Databases<br>Search Screen - Advanced Search<br>Database - CINAHL	4,759
S7	TI "self management" OR AB "self management"	Expanders - Apply equivalent subjects<br>Search modes - Proximity	Interface - EBSCOhost Research Databases<br>Search Screen - Advanced Search<br>Database - CINAHL	19,068
S6	S1 OR S2 OR S3 OR S4 OR S5	Expanders - Apply equivalent subjects<br>Search modes - Proximity	Interface - EBSCOhost Research Databases<br>Search Screen - Advanced Search<br>Database - CINAHL	6,813
S5	TI "inflammatory arthritis" OR AB "inflammatory arthritis"	Expanders - Apply equivalent subjects<br>Search modes - Proximity	Interface - EBSCOhost Research Databases<br>Search Screen - Advanced Search<br>Database - CINAHL	2,057
S4	TI "peripheral spondyloarthritis" OR AB "peripheral spondyloarthritis"	Expanders - Apply equivalent subjects<br>Search modes - Proximity	Interface - EBSCOhost Research Databases<br>Search Screen - Advanced Search<br>Database - CINAHL	67
S3	TI "peripheral spondylarthritis" OR AB "peripheral spondylarthritis"	Expanders - Apply equivalent subjects<br>Search modes - Proximity	Interface - EBSCOhost Research Databases<br>Search Screen - Advanced Search<br>Database - CINAHL	4
S2	(MH "Arthritis, Psoriatic")	Expanders - Apply equivalent subjects<br>Search modes - Proximity	Interface - EBSCOhost Research Databases<br>Search Screen - Advanced Search<br>Database - CINAHL	3,332

S1	TI "psoriatic arthritis" OR "psoriatic arthritis"	Expanders - Apply equivalent subjects<br>Search modes - Proximity	Interface - EBSCOhost Research Databases<br>Search Screen - Advanced Search<br>Database - CINAHL	4,504

Embase <1974 to 2025 February 27>

1	"psoriatic arthritis".ab,ti.	26426
2	exp psoriatic arthritis/	34291
3	"peripheral spondylarthritis".ab,ti.	18
4	"peripheral spondyloarthritis".ab,ti.	414
5	"inflammatory arthritis".ab,ti.	13150
6	1 or 2 or 3 or 4 or 5	47550
7	"self management".ab,ti.	40880
8	exp self care/	112763
9	"self-management".ab,ti.	40880
10	"patient education".ab,ti.	38335
11	exp patient education/	131582
12	"empower*".ab,ti.	61709
13	exp patient empowerment/ or exp empowerment/	16675
14	7 or 8 or 9 or 10 or 11 or 12 or 13	309362
15	6 and 14	828
16	limit 15 to (english language and yr="2010 -Current")	754

Ovid Emcare <1995 to 2025 Week 08>

1	"psoriatic arthritis".ab,ti.	3874
2	exp psoriatic arthritis/	6175
3	"peripheral spondylarthritis".ab,ti.	5
4	"peripheral spondyloarthritis".ab,ti.	62
5	"inflammatory arthritis".ab,ti.	2342
6	1 or 2 or 3 or 4 or 5	8474
7	"self management".ab,ti.	20359
8	exp self care/	57819
9	"self-management".ab,ti.	20359
10	"patient education".ab,ti.	13896
11	exp patient education/	50032
12	"empower*".ab,ti.	34705
13	exp patient empowerment/ or exp empowerment/	18657
14	7 or 8 or 9 or 10 or 11 or 12 or 13	142669
15	6 and 14	197
16	limit 15 to (english language and yr="2010 -Current")	163

EBSCOhost

ROYAL UNITED HOSPITAL
BATH NHS TRUST

Search History/Alerts

Print Search History Retrieve Searches Retrieve Alerts Save Searches / Alerts

Select / deselect all Search with AND Search with OR Delete Searches

Refresh Search Results

Search ID#	Search Terms	Search Options	Actions
S17	S1 AND S13 AND S16	**Limiters -** Publication Date: 20100101-20261231; English Language<br>**Expanders -** Apply equivalent subjects<br>**Search modes -** Proximity	View Results (54) View Details Edit
S16	S2 OR S3 OR S4 OR S5	**Expanders -** Apply equivalent subjects<br>**Search modes -** Proximity	View Results (15,295) View Details Edit
S15	S13 AND S14	**Limiters -** Publication Date: 20100101-20261231; English Language<br>**Expanders -** Apply equivalent subjects<br>**Search modes -** Proximity	View Results (199) View Details Edit
S14	S1 OR S2 OR S3 OR S4 OR S5	**Expanders -** Apply equivalent subjects<br>**Search modes -** Proximity	Rerun View Details Edit
S13	S6 OR S7 OR S8 OR S9 OR S10 OR S11 OR S12	**Expanders -** Apply equivalent subjects<br>**Search modes -** Proximity	Rerun View Details Edit
S12	(MH "Empowerment") OR (MH "Patient Participation")	**Expanders -** Apply equivalent subjects<br>**Search modes -** Proximity	Rerun View Details Edit
S11	AB empower* OR TI empower*	**Expanders -** Apply equivalent subjects<br>**Search modes -** Proximity	Rerun View Details Edit
S10	(MH "Patient Education as Topic+")	**Expanders -** Apply equivalent subjects	Rerun View Details

			**Search modes -** Proximity	Edit	
☐	S9	AB "patient education" OR TI "patient education"	**Expanders -** Apply equivalent subjects<br>**Search modes -** Proximity	Rerun<br>Edit	View Details
☐	S8	(MH "Self-Management")	**Expanders -** Apply equivalent subjects<br>**Search modes -** Proximity	Rerun<br>Edit	View Details
☐	S7	AB "self-management" OR TI "self-management"	**Expanders -** Apply equivalent subjects<br>**Search modes -** Proximity	Rerun<br>Edit	View Details
☐	S6	AB "self management" OR TI "self management"	**Expanders -** Apply equivalent subjects<br>**Search modes -** Proximity	Rerun<br>Edit	View Details
☐	S5	AB "inflammatory arthritis" OR TI "inflammatory arthritis"	**Expanders -** Apply equivalent subjects<br>**Search modes -** Proximity	Rerun<br>Edit	View Details
☐	S4	AB "peripheral spondyloarthritis" OR TI "peripheral spondyloarthritis"	**Expanders -** Apply equivalent subjects<br>**Search modes -** Proximity	Rerun<br>Edit	View Details
☐	S3	AB "peripheral spondylarthritis" OR TI "peripheral spondylarthritis"	**Expanders -** Apply equivalent subjects<br>**Search modes -** Proximity	Rerun<br>Edit	View Details
☐	S2	(MH "Arthritis, Psoriatic")	**Expanders -** Apply equivalent subjects<br>**Search modes -** Proximity	Rerun<br>Edit	View Details
☐	S1	AB "psoriatic arthritis" OR TI "psoriatic arthritis"	**Expanders -** Apply equivalent subjects<br>**Search modes -** Proximity	Rerun<br>Edit	View Details

Search Strategy from ProQuest

28 February 2025 10:00

SEARCH STRATEGY

Set No.	Searched for	Databases	Results
S1	tiab("psoriatic arthritis")	APA PsycInfo®	81
S2	subject(psoriatic arthritis)	APA PsycInfo®	54
S3	tiab("peripheral spondylarthritis")	APA PsycInfo®	0
S4	tiab("peripheral spondyloarthritis")	APA PsycInfo®	0
S5	tiab("inflammatory arthritis")	APA PsycInfo®	90
S6	subject(inflammatory arthritis)	APA PsycInfo®	289
S7	[S1] OR [S2] OR [S3] OR [S4] OR [S5] OR [S6]	APA PsycInfo® These databases are searched for part of your query.	414
S8	tiab("self management")	APA PsycInfo®	11809
S9	subject(self management)	APA PsycInfo®	22515
S10	tiab("self-management")	APA PsycInfo®	11809
S11	tiab("patient education")	APA PsycInfo®	3760
S12	subject(patient education)	APA PsycInfo®	27010
S13	tiab(empower*)	APA PsycInfo®	35429
S14	subject(empowerment)	APA PsycInfo®	12175
S15	[S8] OR [S9] OR [S10] OR [S11] OR [S12] OR [S13] OR [S14]	APA PsycInfo® These databases are searched for part of your query.	89906
S16	[S7] AND [S15]	APA PsycInfo® These databases are searched for part of your query.	19

Database copyright © 2025 ProQuest LLC. All rights reserved.

Terms and Conditions Contact ProQuest
