# Supplementary material for: The self-management support needs of people diagnosed with psoriatic arthritis: a realist review protocol
Source: BMJ Open. 2026 Feb 2;16(2):e110531. doi: 10.1136/bmjopen-2025-110531 (PMC12878256; doi:10.1136/bmjopen-2025-110531)
Supplement: online supplemental file 5 [file bmjopen-16-2-s005.pdf]

TULIPS data extraction form v1

* Required

Record details

1. First Author (surname only) *

2. Year *

3. Country *

4. Record type *

- ◯ Case study
- ◯ Qualitative study
- ◯ Mixed methods study
- ◯ Quantitative study
- ◯ Secondary research
- ◯ National or local policy guidelines
- ◯ Other

5. Source of record *

- ◯ Database search
- ◯ Grey literature search
- ◯ Citation tracking or snowballing
- ◯ Healthcare professional survey
- ◯ Other

Review team

6. Does this record contain complex data which requires team discussion? *

- ◯ Yes
- ◯ No

7. Reviewer(s) *

- ☐ Katie Fishpool
- ☐ Chris Silverthorne
- ☐ Emma Dures
- ☐ Mel Brooke
- ☐ Other

Evidence related to self-management of psoriatic arthritis

8. Content of resources offered

9. Physical and/or psychological wellbeing

10. Previous experiences of health and healthcare

11. Socioeconomic factors

12. Knowledge, experience and/or attitude of healthcare professionals

13. Other factors

This content is neither created nor endorsed by Microsoft. The data you submit will be sent to the form owner.

Microsoft Forms
