# Supplementary material for: The self-management support needs of people diagnosed with psoriatic arthritis: a realist review protocol
Source: BMJ Open. 2026 Feb 2;16(2):e110531. doi: 10.1136/bmjopen-2025-110531 (PMC12878256; doi:10.1136/bmjopen-2025-110531)
Supplement: Supplementary file 6 [file bmjopen-2025-110531.reviewer_comments.pdf]

PEER REVIEW HISTORY

BMJ Open publishes all reviews undertaken for accepted manuscripts. Reviewers are asked to complete a checklist review form and are provided with free text boxes to elaborate on their assessment. These free text comments are reproduced below.

ARTICLE DETAILS

Title (Provisional)

The self-management support needs of people with psoriatic arthritis: a realist review protocol

Authors

Fishpool, Katie; Silverthorne , Christine Anne; Brooke, Mel; McHugh, Neil; Morris, Leah; Ovens, Jason; Smith, Theresa; Tillett, William; Dures, Emma

---

VERSION 1 - REVIEW

Reviewer **1**

Name **Davies, Freya**

Affiliation **Cardiff University, Institute of Primary Care and Public Health**

Date **30-Sep-2025**

COI **None**

Thank you for the opportunity to review your protocol. Here are some suggestions for possible edits.

It could be useful to add to the introduction the rationale for focusing on the initial diagnostic period when self-management support should be an ongoing component of care.

Can the abstract also clarify that this review only looks at the initial diagnostic period and not ongoing development of self-management skills?

Step 2 - can you explain how many papers contributed to the IPT development and reference these?

Search strategy - can you add clarification about any geographical restrictions on including evidence e.g. developed countries only.

Can the full database search strategy be included?

Can you provide a working definition of "self-management" that will be used to define the interventions of interest/exclude others? Similarly can tasks defined as 'self-management' for outcomes be expanded upon?

Can you explain whether any iterative searches are planned? If important IPTs are not covered in the initial search will the search be broadened to attempt to find other supporting evidence (e.g. from later in disease course of from other LTCs)?

Data extraction

Can you list document demographics to be extracted?

Can you describe the quality appraisal process planned?

Data synthesis

Can more details also be given as to how the data for theory testing and refinement will be extracted to ensure that links between context, mechanism and outcome are maintained? What will be the process to develop or refine CMOs using data extracts?

Can you describe how an audit trail will be maintained to show how (and which) data help refine the IPTs?

Can you explain whether any existing substantive theory will be used to inform the review?

Ref 4 is incomplete.

I hope these comments are helpful. Good luck with your review.

VERSION 1 - AUTHOR RESPONSE

Response to comments from Dr. Freya Davies, Cardiff University

Thank you so much for your thoughtful and constructive feedback on our manuscript. I have addressed each comment in detail below.

1. *It could be useful to add to the introduction the rationale for focusing on the initial diagnostic period when self-management support should be an ongoing component of care. Can the abstract also clarify that this review only looks at the initial diagnostic period and not ongoing development of self-management skills?*

Thank you for this helpful point which we have discussed as a team, including with our patient research partner. While the PROMPT study which preceded this work focused on the initial period following diagnosis, this does not feel like a helpful restriction when trying to understand the unmet self-management support needs of people with PsA. PPIE workshops, team discussions and the initial literature review all highlighted that the needs of people with PsA are often not addressed in the early (within 2 years) period, meaning that those needs are still present many years after diagnosis.

Further to this, our patient partner has highlighted that support needs change across the lifespan, with some types of support becoming more relevant as symptoms develop and personal circumstances change. For these reasons, we have agreed to view self-management support as an ongoing need and remove the focus on patients who have recently been diagnosed. To reflect this more accurately in our review we have removed reference to being

newly or recently diagnosed from the title and body of the manuscript.

2. *Step 2 - can you explain how many papers contributed to the IPT development and reference these?*

We are happy to provide further information on these articles to improve transparency of the review process. The details of the articles which contributed to the IPT development, including a summary of their key findings, will now be included as a supplementary file. This is now clearly referenced in the methods section (Step 2).

3. *Search strategy - can you add clarification about any geographical restrictions on including evidence e.g. developed countries only.*

Thank you for this very helpful point. Although we chose not to impose geographical restrictions on our search as we agreed that many experiences of developing self-management skills are universal, the impacts of differing healthcare systems and access to medications are significant and vary between countries. To make sure that this nuance is captured, the country where evidence was generated/collected will be extracted from the included evidence to ensure it is considered in context. This has been included in the data extraction template which is now available as a supplementary file.

4. *Can the full database search strategy be included?*

We are happy to include the full search strategy for each database as a supplementary file.

5. *Can you provide a working definition of "self-management" that will be used to define the interventions of interest/exclude others?*

Thank you for this suggestion. We have been using the definition given in the European Alliance of Associations for Rheumatology (EULAR) published recommendations for the implementation of self-management strategies in patients with inflammatory arthritis (2021) as these felt highly relevant to our studied population. They also highlight that self-management is aimed at achieving independence and that ideally, self-management should be supported by others. The definition, reference and justification have been added to the manuscript in the methods section (Step1).

6. *Similarly can tasks defined as 'self-management' for outcomes be expanded upon?*

As there are many tasks related to self-management, we have now included some examples and supporting references in the manuscript. Alongside the inclusion of a definition of self - management (see #12), we hope that this clarifies the shared understanding of self-management related tasks being used in this review.

14. Can you explain whether any iterative searches are planned?

Thank you for highlighting this point. Iterative searches have not been specifically planned but will be carried out if the included evidence or snowballing/citation searching leads to the identification of terms other than those already searched and that the team agrees may lead to additional evidence. Full details of any further searches carried out and the rationale for these will be provided in the reporting of the completed project.

15. If important IPTs are not covered in the initial search will the search be broadened to attempt to find other supporting evidence (e.g. from later in disease course of from other LTCs)?

We agree that evidence related to self-management across the lifespan should be included (see #8). However we have decided that we will not include evidence related to other health conditions as the purpose of the review is to identify the specific needs of people living with psoriatic arthritis. If IPTs are not supported by the evidence from the literature search, then we would consider this a gap between published evidence and patient/practitioner/researcher perspective that needs investigation. It may be that substantive theory exists for that IPT which relates more generally to IA conditions, but these may not address the specific needs of those with PsA or may not be available to those patients if the resource/intervention/info is not explicitly for that condition.

16. Can you list document demographics to be extracted?

Thank you for this suggestion, a full copy of the data extraction template has now been provided as a supplementary file

17. Can you describe the quality appraisal process planned?

Thank you for this suggestion, we have now included a brief description of the quality appraisal process supported by an appropriate reference in Step 5.

18. Can more details also be given as to how the data for theory testing and refinement will be extracted to ensure that links between context, mechanism and outcome are maintained? What will be the process to develop or refine CMOs using data extracts?
19. Can you describe how an audit trail will be maintained to show how (and which) data help refine the IPTs?

Thank you for these suggestions. The data is being extracted using the five themes relating to the IPTs as a framework (see data extraction template). When data is being synthesised, the contributing evidence will be noted and reported in the final paper. The same process will be followed when data that refines that IPTs comes from PPI contributions or from the HCP survey, so the different types of evidence included are also clearly stated for each theory. This has now been described in greater detail in Step 8 of the methods section.

20. Can you explain whether any existing substantive theory will be used to inform the review?

Thank you for this suggestion. We are aware of significant substantive theory regarding self-management support and inflammatory arthritis, however the preceding PROMPT study highlighted that people diagnosed with PsA have unmet support needs. The aim of this review is therefore to identify the support needs that are specific to PsA and those that are shared with other IA conditions.

21. Ref 4 is incomplete.

Thank you for highlighting this error, the reference has now been completed.
